# Synthesis and Antioxidant Activities of Novel Pyrimidine Acrylamides as Inhibitors of Lipoxygenase: Molecular Modeling and In Silico Physicochemical Studies

**DOI:** 10.3390/molecules29061189

**Published:** 2024-03-07

**Authors:** Michail Saragatsis, Eleni Pontiki

**Affiliations:** Laboratory of Pharmaceutical Chemistry, School of Pharmacy, Faculty of Health Sciences, Aristotle University of Thessaloniki, 54124 Thessaloniki, Greece; michsara262@gmail.com

**Keywords:** pyrimidines, acrylic acids, antioxidants, lipoxygenase, molecular docking

## Abstract

The pyrimidine ring is present in various biomolecules such as DNA and RNA bases, aminoacids, vitamins, etc. Additionally, many clinically used drugs including methotrexate and risperidone contain the pyrimidine heterocyclic scaffold as well. Pyrimidine derivatives present diverse biological activities including antioxidant and anticancer activities and can be considered as privileged scaffolds in drug discovery for the treatment of various diseases. Piperidine pyrimidine amides have gained significant attention due to their enzymatic inhibitory activity. Based on our experience and ongoing investigation on cinnamic acid derivatives, their hybrids and substituted pteridines acting as lipoxygenase inhibitors, antioxidants, anti-cancer, and anti-inflammatory agents a series of novel piperidine pyrimidine cinnamic acids amides have been designed and synthesized. The novel hybrids were studied for their antioxidant and anti-inflammatory potential. They exhibit moderate antioxidant activity in the DPPH assay which may be related to their bulkiness. Moreover, moderate to good lipid peroxidation inhibition potential was measured. With regards to their lipoxygenase inhibitory activity, however, two highly potent inhibitors out of the nine tested derivatives were identified, demonstrating IC_50_ values of 10.7 μM and 1.1 μM, respectively. Molecular docking studies to the target enzyme lipoxygenase support the experimental results.

## 1. Introduction

Oxidative stress is a disturbance in the balance between reactive oxygen species (ROS) production and their deactivation [1,2]. This imbalance provokes tissue damage and induces inflammation [3]. Moreover, ROS including superoxide anion, hydroxyl radical and hydrogen peroxide and their reactive products can damage biological macromolecules, resulting in DNA mutations, lipid peroxidation and protein oxidation [4]. Additionally, they are implicated in arachidonic acid metabolism through cyclooxygenase (COX) and lipoxygenase (LOX) [5,6,7,8]. The latter enzymes catalyze the oxidation of polyunsaturated fatty acids (PUFAs) to the corresponding hydroperoxides, various bioregulatory molecules such as leukotrienes, lipoxins and hepoxylins, hence mediating a variety of pathophysiological diseases such as rheumatoid arthritis, bronchial asthma, psoriasis, cancer and other inflammatory disorders [6,9,10,11]. 

Heterocyclic compounds, due to their structural diversity, play a pivotal role in a range of fields, including agrochemical, veterinary and medicinal chemistry [12]. In particular, nitrogen containing heterocyclics such as pyrimidines present exceptional biopharmacological activities [13]. A considerable number of clinically used drugs such as minoxidil, phenobarbital, primidone, zidovudine, stavudine, 5-flurouracil, methotrexate, imatinib, dasatinib, pazopanib, nilotinib, cytarabine, uramustine, tegafur, risperidone, sulfamethazine and trimethoprim contain the pyrimidine heterocyclic scaffold [14,15]. In recent years, diverse biological activities of pyrimidine derivatives, including anticancer [16,17], antibacterial [18,19,20], antifungal [21], antitubercular [21,22], antimalarial [23], antiviral [24,25,26], analgesic [27], antioxidant [28,29], anti-lipoxygenase [30,31] and anti-inflammatory activities have been reported [32,33]. Thus, the pyrimidine ring can be considered as a promising pharmacophoric scaffold for the synthesis of novel bioactive molecules for the treatment of various diseases.

The group of cinnamic acids (CAs) such as ferulic, caffeic and other phenolic CAs are known to also exert important biological properties including anti-inflammatory and antioxidant [34,35,36], anti-microbial [37,38], anti-tumor [39,40,41], cytoprotective activities ameliorating neuro inflammation in neurogenerative diseases [42,43] and anti-hyperlipidemic activities [44]. They are widely used either as single molecules or in combination with other drugs such as hybrids for improving human health.

**Figure 1:**
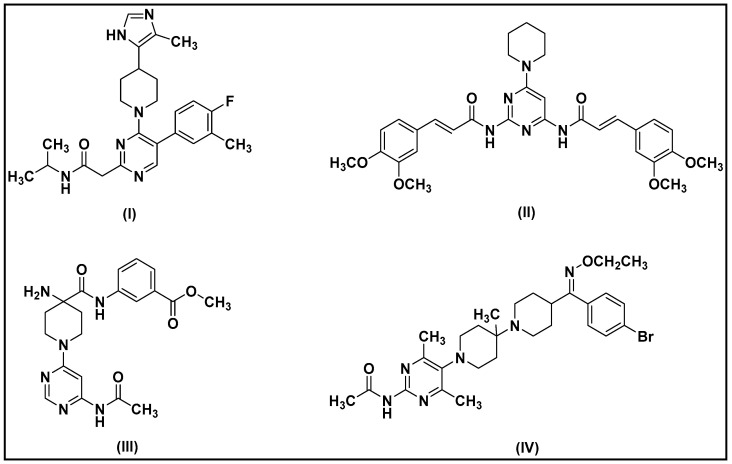
Examples of piperidine pyrimidine amides with inhibitory activity: Inhibitor of sodium hydrogen exchanger-1 (**I**) [49], Inhibitor of in vivo PCA (passive cutaneous anaphylaxis) (**II**) [50], Inhibitor of LIM (Lin-11/Isl-1/Mec-3 domain-containing protein) kinase 1 and LIM kinase 2 (**III**) [51], and Inhibitor of the CCR5 (C-C Chemokine Receptor type 5) (**IV**) [52].

In recent years, piperidine pyrimidine amides are an emerging category of heterocyclic derivatives that have attracted the attention of the scientific community [45,46]. Researchers have focused on piperidine pyrimidine amides due to their various biological activities and potential therapeutic applications. These compounds have been shown to act characterized as inhibitors both for enzymic and non-enzymic targets, making them promising drug candidates (Figure 1) [47,48,49,50,51,52,53]. The combination of these structural features results in chemical properties that make them potentially useful in a variety of applications. 

Hybrid drugs incorporate two different pharmacophores in a single molecule, interacting simultaneously with multiple targets [54,55]. During the last decade, several cinnamic acid derivatives [36,40] and their hybrids (amides combined with different pharmacophores) have been designed and synthesized as potent lipoxygenase inhibitors, antioxidant, anti-cancer and anti-inflammatory agents [56]. Recently our group reported a series of appropriately chemically substituted pteridines exerting antioxidant and anti-inflammatory activities [4] and pyrimidine derivatives as antioxidant and anticancer agents [31]. 

**Figure 2:**
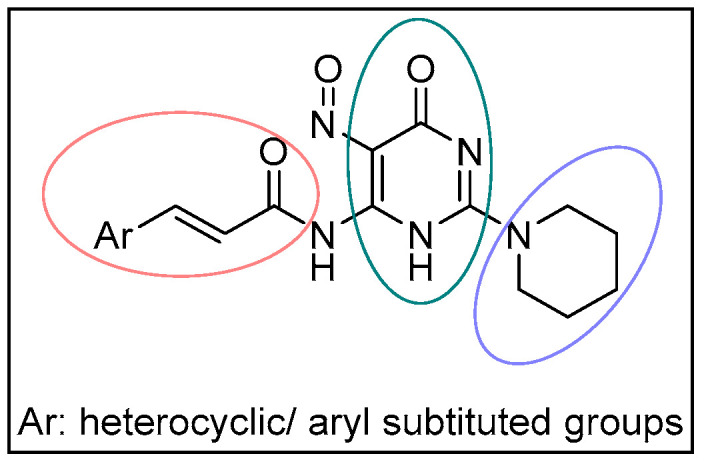
General structure of the newly synthesized hybrids. The encircled portions represent the: (a) the cinnamic acid (red), (b) the pyrimidine (green) and (c) the piperidine (purple) moiety accordingly.

Based on the aforementioned studies, a series of novel hybrid piperidine pyrimidine amides with cinnamic acids has been designed, synthesized and studied as possible antioxidant and anti-inflammatory agents (Figure 2). Molecular docking studies to the target enzyme lipoxygenase have been performed.

## 2. Results and Discussion

### 2.1. Chemistry

**Figure 3:**
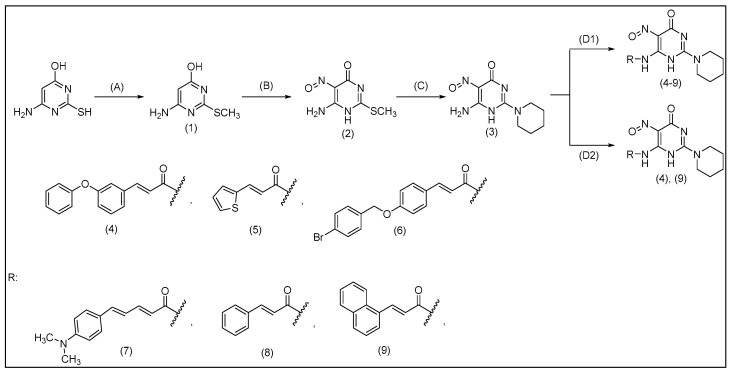
Synthesis of pyrimidine acrylamides (**4**)–(**9**). Reagents and conditions used: (**A**): 6-amino-2-sylfanyl-4(3H)-pyrimidone monohydrate, EtOH, Et_3_N, MeI, 1 h, r.t.; (**B**): 6-amino-(2-methylthio)pyrimidin-4*(3H*)-one (**1**), NaOH/H_2_O, NaNO_2_, gl. CH_3_COOH, 2–3 h, r.t.; (**C**): 6-amino-(2-methylthio) nitrosopyrimidin-4(3H)-one (**2**), EtOH, piperidine, H_2_O, reflux, 6 h; (**D1**): RCOOH, DMF, Et_3_N, 6-amino-5-nitroso-2-(piperidin-1-yl)-pyrimidin-4(1H)-one (**3**), BOP/CH_2_Cl_2_, 30 min-0 °C, 72 h-r.t.; (**D2**): RCOOH, DMF, NHS, EDCI∙HCl, 6-amino-5-nitroso-2-(piperidin-1-yl)-pyrimidin-4(1H)-one (**3**), Et_3_N, 72 h, r.t.

The synthesis of pyrimidine acrylamides was accomplished by an *N*-acylation reaction between 6-amino-5-nitroso-2-(piperidin-1-yl)-pyrimidin-4(*1H*)-one (**3**) and six different carboxylic acids as it is depicted in Figure 3. Two different *N*-acylation methods were used (Figure 3). The first one involved the use of the peptide coupling reagent (dimethylamino)phosphonium hexafluorophosphate (BOP) (Figure 3(D1)) and the second synthetic route proceeded through the formation of the *N*-hydroxysuccinimide active ester which can consequently react very quickly with amines in the presence of 1-Ethyl-3-(3-dimethylaminopropyl)carbodiimide hydrochloride (EDCI∙HCl) (Figure 3(D2)).

Both routes were used to prepare compounds (**4**)–(**9**), whereas only the second method was used to afford compounds (**4**) and (**9**). All compounds are new and they have not been synthesized previously and their structural characterization was based on ^1^H-NMR, ^13^C-NMR, IR, MS (ESI) and elemental analysis data. The most characteristic ^1^H-NMR signals of all compounds are the ones which correspond to the α,β -double bond protons (one doublet signal below δ 7 ppm and second doublet signal above δ 7 ppm with a characteristic coupling constant at approximate 15 Hz), while, on the ^13^C-NMR spectra, the presence of the second carbonyl signal around δ 160 ppm confirms the newly formed amide bond.

### 2.2. In Silico Physicochemical Studies—Computational Analysis

Drug-likeness estimates in a qualitative mode whether a compound can be administered orally with respect to its bioavailability profile. The calculation of properties was carried out through the online Molinspiration software [57]. Molecular weight, topological polar surface area (TPSA), hydrogen-bond donors and acceptors and number of rotational bonds are calculated in order to evaluate the drug-likeness of the compounds and the violation of “Rule of five” (“Lipinski’s rule”) [58]. The calculation of properties includes all the novel hybrids (**4**)–(**9**) and the intermediate compounds (**1**), (**2**) and (**3**), as well, to investigate the role of the amide group on the drug-likeness. 

As it is represented in Table 1, only compound (**6**) violates both molecular weight and LogP properties, which is over 500 and 5 respectively, whereas none of the rest of the compounds present any violation [58]. Compounds (**4**)–(**9**) generally have higher LogP values, indicating their hydrophobic nature. Regarding the antioxidant and anti-inflammatory activity, hydrophobic compounds are more likely to readily cross cell membranes and reach the target site [59]. Oral bioavailability after *per os* administration is closely related with TPSA and the number of rotatable bonds. None of the tested compounds exhibit TPSA values above 140 Å^2^ and number of rotatable bonds more than 10, suggesting a satisfactory *per os* bioavailability [60]. Moreover, the fact that all the compounds have relatively low TPSA values (well below 140 Å^2^), indicate limited polar interactions. However, restricted Blood Brain Barrier (BBB) passage was predicted for all compounds, except (**1**) since for sufficient BBB permeability the TPSA values should be less than 90 [61]. Moreover, the compounds (**4**)–(**9**) have a higher number of rotatable bonds, affecting the molecular flexibility and as a result the possible interaction with the target [62].

### 2.3. Biological Evaluation

Oxidative stress is defined as the imbalance between formation of reactive oxygen species (ROS) and the antioxidant defense mechanisms. There is also an appreciation that increased ROS including superoxide anion, hydroxyl radical, hydrogen peroxide and their reactive products are associated with the pathogenesis of numerous chronic inflammatory conditions including diabetes, cancer, Parkinson’s and Alzheimer’s disease, cardiovascular disease, rheumatoid arthritis and immune dysregulation. Antioxidants are compounds that can prevent or delay oxidation and cell damage, mainly through their ability to scavenge free radicals usually occurring as autoxidation. Antioxidants can be exogenous or naturally occurring and enzymatic or non-enzymatic [63].

Τhe synthesized compounds were studied in vitro to assess: (a) interaction with the stable free radical 2,2-diphenyl-1-picrylhydrazyl radical (DPPH), (b) inhibition of the 2,2′-azobis (2-amidinopropane) dihydrochloride (AAPH)-induced linoleic acid peroxidation and (c) inhibition of soybean lipoxygenase. 

The DPPH assay is employed to evaluate the antioxidant capacity using free radicals for assessing the ability of the synthesized hybrids to serve as hydrogen providers or free-radical scavengers. The DPPH assay is associated with the elimination of DPPH, which would be a stabilized free radical appearing as a lower absorption at 517 nm. The interaction of all the synthesized hybrids with the stable free DPPH was examined at a concentration of 100 μM, both at 20 min and 60 min (Table 2). As a reference compound nordihydroguaretic acid (NDGA), a phenolic lignan strong antioxidant was used [36]. We observed limited reducing ability of the compounds. DPPH reduction activity depends on the ability of the compounds to access the radical site rather than from their chemical properties. The bulkier the compounds are the more difficult to reach into the radical site center. Thus, it is possible that our derivatives due to the substituted pyrimidine ring do not manage to enter the radical center [64].

Oxidative stress has been induced for a variety of antioxidant compounds through redox degradation of hydrogen peroxide or hydroperoxides by metal ions and thermal decomposition of free radical initiators, including hyponitrites, peroxides and azo compounds [65,66]. In this assay, AAPH was selected as the inducer of peroxy radicals and sodium linoleate was utilized as substrate to peroxidation. AAPH is a water-soluble azo compound having the ability to directly generate peroxyl radicals at room temperature and at a constant and reproducible rate without generating H_2_O_2_ (hydrogen pexoxide) as an intermediate [67,68,69]. During this experiment, sodium linoleate was transformed into 13-hydroperoxy-linoleic acid, and this transformation was measured at 234 nm. Consequently, any variation in absorbance indicates the possible antioxidant effect of the synthesized derivatives compared to the reference compound, Trolox (Table 2). According to the biological evaluation, the tested compounds (**4**–**7**) showed high activity (71–82%) apart from compound (**6**) which presented medium activity 30%. Compounds (**8**) and (**9**) were found to be inactive. Compound (**7**), the pyrimidine derivative of *(2E,4E)-*5-(4-(dimethylamino)phenyl)penta-2,4-dienoic acid, exhibited the highest activity (82%) followed by compound (**5**), the pyrimidine derivative of 3-(2-thienyl)acrylic acid (78%). It may be deduced that the presence of an additional double bond favors the activity due to the resemblance to the natural substrate.

Lipoxygenases (LOX) play a pivotal role in the regulation of inflammatory responses by generating potent proinflammatory mediators such as leukotrienes or anti-inflammatory substances known as lipoxins. The plant derived LOX (soybean) has been extensively utilized in a variety of studies [41] and could be characterized as a representative analogous model to human 5-LOX due to its satisfactory homology [42]. Plant LOX has linoleic acid as a substrate. As an indicative study for anti-inflammatory activity, the synthesized compounds were tested for their ability to inhibit soybean lipoxygenase (Table 2). The most potent pyrimidine derivatives proved to be compound (**9**) presenting an IC_50_ = 1.1 μM and compound (**5**) presenting an IC_50_ = 10.7 μM. Compound (**9**) is a promising lipoxygenase inhibitor and its calculated IC_50_ value is close to the activity to NDGA, a well-known lipoxygenase inhibitor, used in this study as reference compound. The other final pyrimidine derivatives (**4**, **6**–**8**) presented moderate activity (35–49%) while the starting material and the intermediates did not present any activity. 

### 2.4. Computational Analysis—Molecular Docking on Soybean Lipoxygenase 

Docking studies were performed for the intermediates as well for the final acrylamides. Soybean lipoxygenase-1 (PDB ID: 3PZW) was chosen to comply with the biological protocol. It is well known that lipoxygenases are hyperoxidases containing a ‘‘non-heme’’ iron per molecule. They catalyze the reaction of molecular oxygen with free and esterified polyunsaturated fatty acids containing a (*1Z,4Z*)-penta-1,4-diene system turning them into the corresponding hydroperoxides. According to recent literature, lipoxygenases in addition to the substrate compass other possible allosteric binding sites [70,71,72]. Docking studies have been conducted to investigate potential binding modes to both the active site and the entire protein encompassing all other sites. The synthesized compounds do not appear to have the ability to enter into the binding site of the enzyme. These findings are supported by previous publications [71,73,74]. Table 3 presents the binding affinity values for all the compounds, expressed in kcal/mol. A lower docking score indicates a more favorable binding affinity. 

The intermediate compounds (**1**), (**2**) and (**3**) did not exhibit good docking scores following the experimental results. From the biological evaluation, it can be concluded that the most active derivatives are compounds (**5**) and (**9**), presenting AutoDock Vina scores of −8.7 and −10.6 kcal/mol, respectively. There is not 100% correlation between docking scores and IC_50_ values. Docking affinity values are based on algorithms and scoring function calculations while on the other hand IC_50_ values are calculated experimentally. Docking provides putative binding mode of the ligand with the protein instead IC_50_ provides the value for inhibitory activity. 

**Figure 4:**
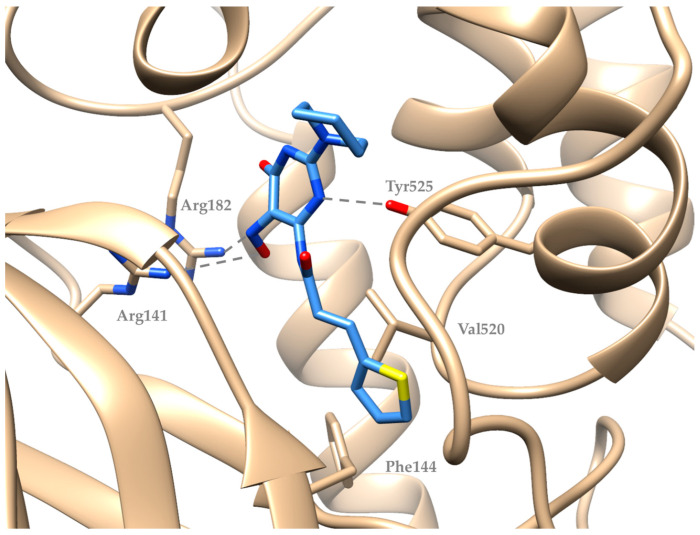
Preferred docking pose of compound (**5**) bound to soybean lipoxygenase (PDB ID: 3PZW).

**Figure 5:**
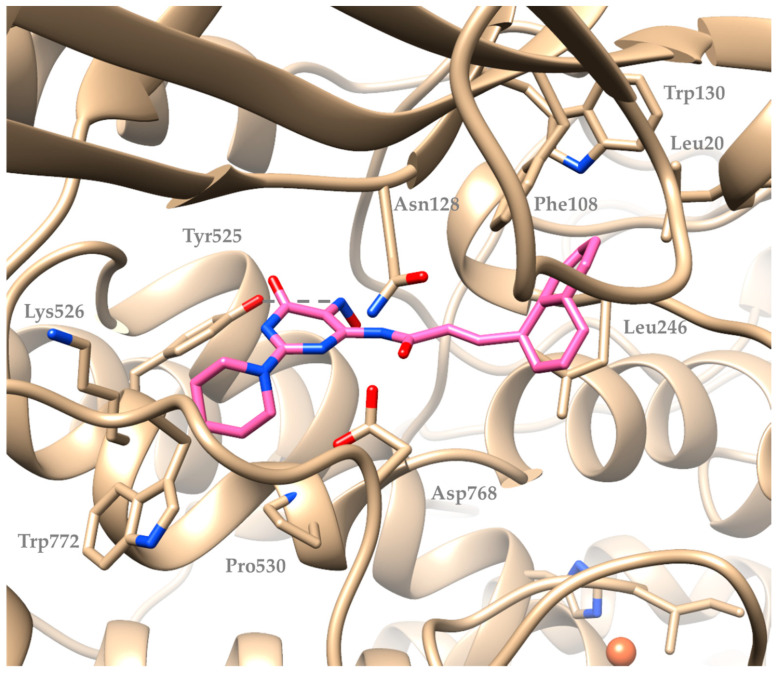
Preferred docking pose of compound (**9**) bound to soybean lipoxygenase (PDB ID: 3PZW).

**Figure 6:**
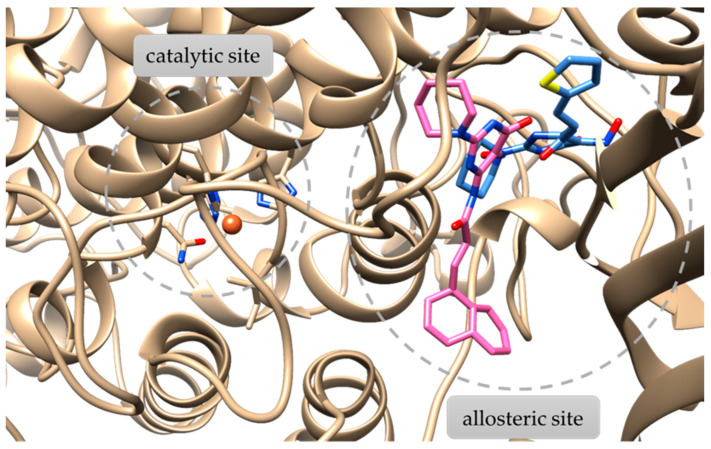
Preferred binding poses of compounds (**5**) and (**9**) bound to soybean lipoxygenase (PDB ID: 3PZW). The catalytic and the allosteric sites are highlighted with grey dashed lines. The orange sphere at the catalytic site represents the iron atom.

Compound (**5**) seems to interact with soybean lipoxygenase with hydrophobic interactions with Phe144 and Val520 and hydrogen bonds with Arg141, Arg182, and Tyr525 residues (Figure 4). Compound (**9**), being the most active, develops hydrogen bonds with Asn128 and Tyr525, hydrophobic contacts with Leu20, Trp130, Leu246, Lys526, Pro530, Trp772 and π-stacking with Phe108 (Figure 5). Figure 6 describes the binding mode of the two novel derivatives with the enzyme. They seem to interact in a different mode but the hydrogen bond with Tyr525 seems to be important for their binding mode. It is well known that a significant number of LOX inhibitors act as antioxidants or scavengers of free radicals [36] and lipoxygenase reaction takes place through a carbon-centered radical on a lipid chain. Probably, compounds (**5**) and (**9**) interact with SLOX by expanding into the hydrophobic pocket, hindering natural substrates from reaching the active site and hence inhibiting the oxidation cycle by soybean lipoxygenase. 

## 3. Experimental Section

### 3.1. Materials and Instruments

Analytical grade chemical and biochemical reagents have been utilized and solvents were supplied from commercial sources (Sigma, St. Louis, MO, USA, Merck, Merck KGaA, Darmstadt, Germany, Fluka Sigma–Aldrich Laborchemikalien GmbH, Hannover, Germany, Alfa Aesar, Karlsruhe, Germany) and used without further purification. Sodium linoleate, soybean lipoxygenase and 2,2-azobis-(2- amidinopropane) dihydrochloride (AAPH) were obtained from Sigma Chemical, Co. (St. Louis, MO, USA). Also, the 1,1-diphenyl-2-picrylhydrazyl (DPPH) and Nordihydroguairetic acid (NDGA) were acquired from the Aldrich Chemical Co. (Milwaukee, WI, USA). 

A MEL-Temp II (Lab. Devic-es, Holliston, MA, USA) was used to determine the melting points of the solid compounds. The in vitro tests have been carried out using UV–VIS spectra and were recorded on a Perkin-Elmer 554 double-beam spectrophotometer (Perkin-Elmer Corporation Ltd., Lane Beaconsfield, Bucks, UK) and UV-1700 UV-Visible spectrophotometer PharmaSpec (Shimadzu, Duisburg, Germany). Infrared spectra were obtained with Perkin-Elmer 597 spectrophotometer (Perkin-Elmer Corporation Ltd., Lane Beaconsfield, Bucks, UK). The spectra were recorded using either Nujol or KBr pellets.

A Bruker AM 300 spectrometer (Bruker Analytische Messtechnik GmbH, Rheinstetten, Germany) was used to record the ^1^H Nucleic Magnetic Resonance (NMR) spectra at 300 MHz and the ^13^C-NMR spectra at 75.5 MHz either in CDCl_3_ or DMSO using tetramethylsilane as an internal standard unless otherwise stated. Chemical shifts are denoted in parts per million (ppm), while the coupling constants (*J* values) are expressed in Hz. Mass spectra were acquired using a LC-MS 2010 EV Shimadzu (Shimadzu, Kiyoto, Japan), with MeOH as the solvent. The elemental analyses for carbon (C) and hydrogen (H) resulted in values that closely matched the theoretical values within an acceptable range of ±0.4%. The above analyses were conducted using a Perkin-Elmer 240B CHN analyzer, provided by Perkin-Elmer Corporation Ltd., located in Lane Beaconsfield, Bucks, UK. The progress of the reactions was monitored by thin layer chromatography on 5554 F254 silica gel/TLC cards, which were purchased from Merck and Fluka Chemie GmbH (Buchs, Steinheim, Switzerland). 

### 3.2. Chemistry General Procedure

#### 3.2.1. Synthesis of 6-amino-2-(methylthio)-pyrimidin-4-ol (**1**)

6-amino-2-sylfanyl-4 *(3H)*-pyrimidone monohydrate (1 eq.) was suspended in a mixture of water and ethanol (7 mL). Triethylamine (d = 0.725 g/mL) (2 eq.) was added and the solution became clear. Methyliodide (d = 2.28 g/mL) (1.1 eq.) was added to the stirring solution. Within a few minutes an exothermic reaction started with formation of a colourless precipitate. Stirring was continued for 30 min–1 h. The progress of the reaction was monitored with TLC. The mixture was cooled to 4 °C (overnight) and the precipitate was filtered, washed with water and diethylether to afford the title compound. The compound was collected, dried in vacuum and characterized.

Yield: 83.4%, R_f_ (20% Methanol/Chloroform) = 0.61, Melting point: 267–268 °C.

The compound is mentioned in the bibliography, following the procedure outlined in reference [75].

#### 3.2.2. Synthesis of 6-amino-2-(methylthio)-5-nitrosopyrimidin-4(3H)-one (**2**)

6-amino-(2-methylthio)pyrimidin-4*(3H)-*one (**1**) (1 eq.) was dissolved in a solution of sodium hydroxide (1 eq.) in water at room temperature. Additional sodium hydroxide was added till complete dissolution. Then, a solution of sodium nitrite (1.5 eq.) was added. The yellow solution was acidified by the gradual addition of glacial acetic acid (2.2 eq.). A white precipitate formed almost immediately. The progress of the reaction was monitored with TLC. Stirring was continued for 2–3 h, till starting material disappeared. The mixture was filtrated, washed with water and diethylether and the precipitate was collected, dried in vacuum and characterized spectroscopically.

Yield: 65%. R_f_ (20% Methanol/Chloroform) = 0.66. Melting point: 247–248 °C. ^1^H NMR (300 MHz, DMSO): δ 2.54 ppm (s, 3H), 9.00 ppm (brs, 1H), 11.26 ppm (brs, 1H), 12.67 ppm (brs, 1H).

The compound is mentioned in the bibliography, following the procedure outlined in reference [76].

#### 3.2.3. Synthesis of 6-amino-5-nitroso-2-(piperidin-1-yl)-pyrimidin-4(1H)-one (**3**)

To 6-amino-(2-methylthio) nitrosopyrimidin-4(*3H*)-one (**2**) (1 eq.) in absolute ethanol (1.5 mL), piperidine (d = 0.862 g/mL) (4 eq.) was added. The mixture was heated under reflux for 5 h. Water (1.5 mL) was then added and the mixture was heated under reflux for a further 1 h. The progress of the reaction was monitored with TLC. The solution was immediately and rapidly cooled. No formation of precipitate, so the solution was evaporated and recrystallized from ethanol. By leaving overnight a reddish precipitate was formed, which was collected with filtration, dried in vacuum and characterized spectroscopically.

Yield: 78.3%. R_f_ (20% Methanol/Chloroform) = 0.83. Melting point: 241–243 °C. ^1^H NMR (300 MHz, CDCl_3_): δ 1.79–1.87 ppm (m, 6H), 3.06–3.10 ppm (m, 4H), 5.56 ppm (d, *J* = 5.6 Hz, 1H) 10.88 ppm (s, 1H).

The compound is mentioned in the bibliography, following the procedure outlined in reference [77].

#### 3.2.4. Synthesis of Substituted Acrylamides

Method A: The acid (0.5 eq.) was dissolved in 1 mL of Dimethylformamide (DMF) and 0.5 eq. of triethylamine. The solution was cooled in an ice-water bath and 0.5 eq. of 6-amino-5-nitroso-2-(piperidin-1-yl)-pyrimidin-4(*1H*)-one (**3**) was added, followed by a solution of 0.5 eq. of benzotriazol-1-yloxytris(dimethylamino)phosphonium hexafluorophosphate (BOP) in 1 mL of dichloromethane. The mixture was stirred at 0 °C for 30 min and then at room temperature for 72 h. The progress of the reaction was monitored with TLC. Dichloromethane was removed under reduced pressure and 8 mL of water was added in the solution. The products were extracted with ethylacetate and the combined organic layers were washed with 1N HCl acid (2 times), water, 1M NaHCO_3_ (2 times) and water. The organic extract dried over Magnesium Sulfate, filtered and evaporated under vacuum. Each compound was purified either by recrystallization or column chromatography and characterized spectroscopically (Supplementary Information).

Method B: The acid (1 eq.) was dissolved in 1.5 mL of Dimethylformamide (DMF). *N*-hydroxysuccinimide (NHS) (1.7 eq.) and 1-Ethyl-3-(3-dimethylaminopropyl)carbodiimide hydrochloric (EDCI∙HCl) (1.7 eq.) were added to the above solution and the reaction mixture was stirred at room temperature and was monitored by TLC until the formation of the activated ester intermediate. Then, 6-amino-5-nitroso-2-(piperidin-1-yl)-pyrimidin-4(1H)-one (**3**) (1 eq.) and Triethylamine (d = 0.73 g/mL) (0.4 eq.) were added to the reaction mixture and was stirred at room temperature for 72 h. The progress of the reaction was monitored with TLC till the starting materials were consumed. This was followed by quenching with Water (10 mL) and the mixture was extracted with Ethylacetate (3 × 10 mL). The combined organic layers were washed with Water (3 × 20 mL) and brine, dried over Magnesium Sulfate, filtered off and concentrated under vacuum and characterized spectroscopically (Supplementary Information).

#### (*E)-N*-(5-nitroso-6-oxo-2-(piperidin-1-yl)-3,6-dihydropyrimidin-4-yl)-3-(3-phenoxyphenyl)acrylamide (**4**)

The compound (**4**) was recrystallized from Ethylacetate/Petroleum Ether (EA/PE). Yield (Method A): 55%, Yield (Method B): 53%. R_f_ (10% Methanol/Chloroform) = 0.79. Melting Point: 193–195 °C. ^1^H NMR (300 MHz, CDCl_3_): δ 1.53–1.63 ppm (m, 6H), 3.54–3.57 ppm (m, 4H), 6.79 ppm (d, *J* = 15.5 Hz, 1H), 6.89–6.97 ppm (m, 4H), 7.08 ppm (dt, *J* = 14.8, 4.7 Hz, 2H), 7.20 ppm (t, *J* = 9.7 Hz, 2H), 7.29 ppm (s, 1H), 7.53 ppm (d, *J* = 15.5 Hz, 1H). ^13^C NMR (75 MHz, CDCl_3_): δ 24.8 ppm, 26.3 ppm, 29.8 ppm, 43.8 ppm, 117.8 ppm, 118.6 ppm, 119.0 ppm, 119.4 ppm, 119.6 ppm, 119.9 ppm, 123.1 ppm, 123.6 ppm, 130.0 ppm, 130.1 ppm, 130.2 ppm, 137.5 ppm, 141.7 ppm, 157.1 ppm, 157.7 ppm, 163.6 ppm, 165.3 ppm. ESI-MS *m*/*z* (positive): [M + H + Na + MeOH + Pyrimidinone]^+^ calculated for C_29_H_28_N_9_O_7_Na 637; found 637, [M + H − Piperidine]^+^ calculated for C_19_H_14_N_4_O_4_ 362; found 362, [Μ + H − Pyrimidinone]^+^ calculated for C_20_H_22_NO_2_ 308; found 308. IR (KBr) (cm^−1^): 3050–2940, 1720, 1640, 1460, 1280. Anal. C, H, N. Calc %: (C_24_H_23_N_5_O_4_) C: 64.71, H: 5.20, N: 15.72, Found %: C: 64.78, H: 5.31, N: 15.65.

#### (*E)-N-(*5-nitroso-6-oxo-2-(piperidin-1-yl)-3,6-dihydropyrimidin-4-yl)-3-(thiophen-2-yl)acrylamide (**5**)

The compound (**5**) was separated by Column Chromatography (50% Petroleum Ether/Ethylacetate). Yield (Method A): 84%. R_f_ (20% Methanol/Chloroform) = 0.77. Melting Point: 106–108 °C. ^1^H NMR (300 MHz, CDCl_3_): δ 1.56–1.71 ppm (m, 6H), 3.57–3.61 ppm (m, 4H), 6.69 ppm (d, *J* = 15.1 Hz, 1H), 7.01 ppm (dd, *J* = 5.0, 3.5 Hz, 1H), 7.18 ppm (d, *J* = 3.5 Hz, 1H), 7.28 ppm (d, *J* = 5.1 Hz, 1H), 7.76 ppm (d, *J* = 15.1 Hz, 1H). ^13^C NMR (75 MHz, CDCl_3_): δ 24.7 ppm, 26.2 ppm, 29.7 ppm, 45.2 ppm, 116.5 ppm, 120.3 ppm, 126.9 ppm, 127.9 ppm, 129.8 ppm, 135.1 ppm, 140.7 ppm, 148.8 ppm, 153.8 ppm, 163.8 ppm, 165.2 ppm. ESI-MS *m*/*z* (positive): [M + Na + piperidine]^+^ calculated for C_21_H_26_N_6_O_3_SNa 465; found 465, [M + H − piperidine]^+^ calculated for C_11_H_8_N_4_O_3_S 276; found 276, [M + H − pyrimidinone]^+^ calculated for C_12_H_16_NOS 222; found 222. IR (KBr) (cm^−1^): 3245, 3010, 1715, 1680, 1260. Anal. C, H, N. Calc %: (C_16_H_17_N_5_O_3_S) C: 53.47, H: 4.77, N: 19.49, Found %: C: 53.60, H: 4.70, N: 19.65.

#### (*E)-*3-(4-((4-bromobenzyl)oxy)phenyl)-N-(5-nitroso-6-oxo-2-(piperidin-1-yl)-3,6-dihydropyrimidin-4-yl)acrylamide (**6**)

The compound (**6**) was recrystallized twice from Ethylacetate/Petroleum Ether. Yield (Method A): 22%. R_f_ (Petroleum Ether/Ethylacetate 1:1) = 0.56. Melting Point: 130–132 °C. ^1^H NMR (300 MHz, CDCl_3_): δ 1.59–1.68 ppm (m, 6H), 3.57–3.66 ppm (m, 4H), 5.03 ppm (s, 2H), 6.78 ppm (d, *J* = 15.3 Hz, 1H), 6.93 ppm (d, *J* = 8.4 Hz, 2H), 7.30 ppm (d, *J* = 7.8 Hz, 2H), 7.46 ppm (d, *J* = 8.1 Hz, 2H), 7.51 ppm (d, *J* = 7.8 Hz, 2H) 7.60 ppm (d, *J* = 15.3 Hz, 1H). ^13^C NMR (75 MHz, CDCl_3_): δ 24.8 ppm, 26.4 ppm, 45.6 ppm, 69.6 ppm, 115.4 ppm, 115.8 ppm, 122.2 ppm, 124.9 ppm, 129.2 ppm, 129.4 ppm, 132.0 ppm, 133.5 ppm, 136.0 ppm, 142.1 ppm, 144.2 ppm, 146.0 ppm, 155.6 ppm, 159.9 ppm, 165.9 ppm. ESI-MS *m*/*z* (positive): [M + H − pyrimidinone]^+^ calculated for C_21_H_23_BrNO_2_ 400; found 400. IR (KBr) (cm^−1^): 3050–2940, 1730, 1610, 1515, 1250, 1230. Anal. C, H, N. Calc %: (C_25_H_24_BrN_5_O_4_) C: 55.77, H: 4.49, N: 13.01, Found %: C: 55.69, H: 4.57, N: 13.12.

#### (2*E,4E)-*5-(4-(dimethylamino)phenyl)-N-(5-nitroso-6-oxo-2-(piperidin-1-yl)-3,6-dihydropyrimidin-4-yl)penta-2,4-dienamide (**7**)

The compound (**7**) was recrystallized twice from Ethylacetate/Petroleum Ether. Yield (Method A): 24%. R_f_ (Petroleum Ether/Ethylacetate 1:1) = 0.74. Melting Point: 175–177 °C. ^1^H NMR (300 MHz, CDCl_3_): δ 1.67–1.77 ppm (m, 6H), 3.12 ppm (s, 6H), 3.89–3.98 ppm (m, 4H), 7.54–7.59 ppm (m, 3H), 7.79 ppm (t, *J* = 7.9 Hz, 1H), 8.05 ppm (dd, *J* = 20.3, 8.4 Hz, 3H), 8.59 ppm (d, *J* = 16.6 Hz, 1H). ^13^C NMR (75 MHz, CDCl_3_): δ 24.3 ppm, 25.8 ppm, 31.1 ppm, 43.4 ppm, 45.6 ppm, 114.7 ppm, 115.9 ppm, 117.1 ppm, 125.4 ppm, 126.5 ppm, 129.1 ppm, 132.7 ppm, 133.4 ppm, 143.7 ppm, 152.9 ppm, 160.5 ppm, 163.0 ppm, 165.3 ppm. ESI-MS *m*/*z* (positive): [M + H + Na + piperidine + pyrimidinone]^+^ calculated for C_31_H_38_N_11_O_5_Na 667; found 667, [M + H − NO]^+^ calculated for C_22_H_27_N_5_O_2_ 393; found 393, [M+K-piperidine]^+^ calculated for C_17_H_16_N_5_O_3_K 377; found 377, [M + Na − piperidine]^+^ calculated for C_17_H_16_N_5_O_3_Na 361; found 361, [M + K − pyrimidinone]^+^ calculated for C_18_H_25_N_2_OK 324; found 324. IR (KBr) (cm^−1^): 3270, 3050–2940, 1705, 1615, 1520, 1210. Anal. C, H, N. Calc %: (C_22_H_26_N_6_O_3_) C: 62.54, H: 6.2, N: 19.89, Found %: C: 62.6, H: 6.26, N: 19.92.

#### *N*-(5-nitroso-6-oxo-2-(piperidin-1-yl)-3,6-dihydropyrimidin-4-yl)-3-phenylacrylamide (**8**)

Yield (Method A): 22%. R_f_ (Petroleum Ether/Ethylacetate 1:1) = 0.57. Melting Point: 104–106 °C. ^1^H NMR (300 MHz, CDCl_3_): δ 1.60–1.69 ppm (m, 6H), 3.63 ppm (t, *J* = 1.5 Hz, 4H), 6.89 ppm (d, *J* = 15.5 Hz, 1H), 7.34–7.38 ppm (m, 3H), 7.52 ppm (d, *J* = 7.5 Hz, 2H), 7.65 ppm (d, *J* = 15.5 Hz, 1H). ^13^C NMR (75 MHz, DMSO): δ 24.2 ppm, 25.5 ppm, 26.6 ppm, 42.7 ppm, 46.2 ppm, 118.6 ppm, 119.9 ppm, 125.5 ppm, 128.0 ppm, 128.8 ppm, 129.5 ppm, 135.3 ppm, 141.2 ppm, 144.8 ppm, 164.3 ppm, 179.3 ppm. ESI-MS *m*/*z* (positive): [M + H − piperidine]^+^ calculated for C_13_H_10_N_4_O_3_ 270; found 270. IR (KBr) (cm^−1^): 3250, 3050–2940, 1740, 1620, 1505, 1280. Anal. C, H, N. Calc %: (C_18_H_19_N_5_O_3_) C: 61.18, H: 5.42, N: 19.82, Found %: C: 61.06, H: 5.50, N: 19.94.

#### (*E)-*3-(naphthalen-1-yl)-N-(5-nitroso-6-oxo-2-(piperidin-1-yl)-3,6-dihydropyrimidin-4-yl)acrylamide (**9**)

The compound (**9**) was isolated by Preparative Chromatography with the use of Ethylacetate/Petroleum ether 2:1 (EA/PE 2:1). Yield (Method A): 15%, Yield (Method B): 38%. R_f_ (30% Petroleum Ether/Ethylacetate) = 0.78. Melting Point:—(Oil). ^1^H NMR (300 MHz, CDCl_3_): δ 1.62–1.71 ppm (m, 6H), 3.67–3.72 ppm (m, 4H), 6.96 ppm (d, J = 15.8 Hz, 1H), 7.45–7.57 ppm (m, 3H), 7.71 ppm (d, *J* = 6.4 Hz, 1H), 7.86 ppm (d, *J* = 8.2 Hz, 2H), 8.24 ppm (d, *J* = 7.8 Hz, 1H), 8.50 ppm (d, *J* = 16.2 Hz, 1H). ^13^C NMR (75 MHz, CDCl_3_): δ 24.8 ppm, 29.8 ppm, 43.6 ppm, 47.2 ppm, 120.9 ppm, 124.0 ppm, 124.6 ppm, 125.5 ppm, 126.3 ppm, 126.7 ppm, 128.7 ppm, 129.8 ppm, 131.6 ppm, 133.4 ppm, 133.7 ppm, 137.9 ppm, 139.7 ppm, 148.6 ppm, 152.0 ppm, 162.1 ppm, 165.4 ppm. ESI-MS *m*/*z* (positive): [2 × M − piperidinepyrimidinone − NO]^+^ calculated for C_35_H_29_N_4_O_3_ 553; found 553, [M + H − piperidine]^+^ calculated for C_13_H_10_N_4_O_3_ 352; found 352. IR (KBr) (cm^−1^): 3370, 3100, 1710, 1660, 1555, 1240. Anal. C, H, N. Calc %: (C_22_H_21_N_5_O_3_) C: 65.50, H: 5.25, N: 17.36, Found %: C: 65.62, H: 5.38, N: 17.22.

### 3.3. Biological Assays

Each in vitro assay was carried out at least three times and the standard deviation of absorbance observed to be less than 10% of the mean. A stock solution (1% of tested compound diluted in DMSO under sonification) was prepared in order to take place the in vitro assays.

#### 3.3.1. Interaction of the Novel Compounds with the Stable Radical 1,1-diphenyl-picrylhydrazyl (DPPH)

A particular volume of the tested compounds (0.05 mM and 0.1 mM final concentrations) dissolved in DMSO and they were incubated with a solution of DPPH in absolute ethanol in absence of light. The absorbance was recorded at 517 nm after 20 and 60 min at room temperature (Table 2). The test follows our previous published methods [31,73].

#### 3.3.2. Inhibition of Linoleic Acid Lipid Peroxidation

The water-soluble compound 2,2’-azobis(2-amidinopropane) dihydrochloride (AAPH) was used as a free radicals initiator in order to start the lipid peroxidation of sodium linoleate. A total of 930 μL of a phosphate buffer solution (0.05 M, pH = 7.4), 10 μL of the sodium linolate solution (16 mM in a buffer solution of Tris-HCl pH = 9) and 10 μL of the tested compounds (stock solutions 100 μΜ) were mixed and the oxidative procedure started after the addition of 50 μL AAPH at 37 °C under air conditions. The conjugated diene was monitored at 234 nm. Compounds with antioxidant properties decrease the rate of oxidation (Table 2). The assay was performed in accordance with previous published methods. Trolox was used as a reference compound [36].

#### 3.3.3. Soybean Lipoxygenase Inhibition Assay

Sodium linoleate (0.1 mM), 0.2 mL of soybean lipoxygenase solution (1/9 × 10**^−^**^4^ *w*/*v* in saline) and 10 μL from a stock solution (10 mM) of tested compounds in DMSO were incubated at room temperature. The conjugated diene was monitored at 234 nm. Nor-dihydroguaretic acid (NDGA) was used as a reference compound. Several concentrations were used for the determination of IC_50_ values (Table 2). The experiment was performed according to previous publications [36].

### 3.4. Computational Analysis—Molecular Docking on Soybean Lipoxygenase

The visualization and preprocessing of the protein (PDB ID: 3PZW) was conducted using UCSF Chimera (version 1.15) [78]. Water molecules and extraneous crystallographic material were removed with Chimera. Modeller (v. 10.3) [79] was applied to add missing residues (Met1-Phe2-Ser3-Ala4-Gly5, Glu21-Val22-Asn23-Pro24-Asp25-Gly26-Ser27-Ala28-Val28-Asp29, Ile117-Ser118-Asn119-Gln120). Hydrogen atoms and charges were added with AmberTools [80,81]. The charge on iron was adjusted to +2.0 applying the 12–6 LJ nonbonded model [82]. Histidines His499, His504 and His690 coordinating iron were set neutral (δ-nitrogen protonated state). TIP3P explicit water model was used. The size of the box has a minimal distance of 12 Å between the solute and the edge of the box.

Furthermore, the generation and minimization of ligand 3D coordinates were carried out using Open Babel (version 3.1.1) [83] with the MMFF94 force field [84]. ACPYPE [85] was utilized to produce the ligand topologies and parameters using AnteChamber (AmberTools v. 22.10) [86]. The energy minimization of the protein was performed using GROMACS (v. 4.6.5) [87]. AutoDock Vina (v. 1.2.3) [88,89] was used to dock the ligands into the protein. This was achieved by configuring a grid box center x = 1.35 Å, y = 14.3 Å and z = −34.60 Å and size of x = 100 Å, y = 70 Å and z = 70 Å. Moreover, the exhaustiveness parameter was configured with the value of 10 and 20 docking modes were generated as maximum output. UCSF Chimera utilized to analyze the docking results.

## 4. Conclusions

Six novel pyrimidine acrylamides were designed, synthesized and evaluated for their antioxidant and anti-inflammatory activities. The majority of the synthesized compounds (except from compound **6**) followed the Lipinski rule of five; thus, the compounds may have good oral bioavailability. Half of the newly synthesized compounds, (**4**), (**5**) and (**7**), are potent lipid peroxidation inhibitors. However, in the DPPH assay, the majority of the compounds presented limited reducing activity (2–25%). Furthermore, the most potent LOX inhibitors proved to be the derivatives (**9**) and (**5**) with an IC_50_ value equal to 1.1 μM and 10.7 μM respectively, while most of the compounds (**4**, **6**–**8**) presented moderate inhibitory activity. Docking studies of the most active derivatives showed that development of hydrogen bond with Tyr525 seems to be important. Compound (**9**) is a promising lipoxygenase inhibitor presenting IC_50_ value close to NDGA a well-known lipoxygenase inhibitor. Compound (**9**) could be used in the future as lead compound for the synthesis of novel antioxidant derivatives with anti-lipoxygenase activity.

## Figures and Tables

**Table 1:** Drug-likeness/physicochemical properties of the synthesized compounds (1)–(9).

Compound	No of Atoms	milogP ^a^	MW ^b^	No of OH and NH ^c^	No of O and N ^d^	No of Violations	TPSA ^e^	No of Rotatable Bonds ^f^	Volume ^g^
(**1**)	10	1.22	157.20	3	4	0	72.03	1	129.73
(**2**)	12	0.58	186.20	3	6	0	101.21	2	144.66
(**3**)	16	0.98	223.24	3	7	0	104.45	2	195.92
(**4**)	33	4.75	445.48	2	9	0	116.76	7	395.24
(**5**)	25	2.74	359.41	2	8	0	107.53	5	305.56
(**6**)	35	5.48	538.40	2	9	2	116.76	8	429.93
(**7**)	31	3.65	422.49	2	9	0	110.76	7	388.17
(**8**)	26	3.02	353.38	2	8	0	107.53	5	314.85
(**9**)	30	4	403.44	2	8	0	107.53	5	358.84

^a^ Logarithm of partition coefficient based on group contributions (milogP); ^b^ molecular weight; ^c^ number of hydrogen bond donors; ^d^ number of hydrogen bond acceptors; ^e^ topological polar surface area (TPSA); ^f^ number of rotatable bonds; ^g^ molecular volume.

**Table 2:** Antioxidant and Anti-inflammatory activity of the developed compounds based on DDPH, APPH and soybean LOX inhibition in vitro assays.

Compd.	% Interaction with the Stable Free Radical DPPH, at 100 μM 20 min	% Interaction with the Stable Free Radical DPPH, at 100 μM 60 min	% Inhibition of AAPH-Induced Linoleic Acid Peroxidation at 100 μM	% Inhibition of LOX 100 μM or IC_50_ (μM) *
(**1**)	na	na	na	na
(**2**)	18	9	na	na
(**3**)	18	19	na	na
(**4**)	14	15	71	35%
(**5**)	22	3	78	10.7 μM
(**6**)	21	na	30	38%
(**7**)	23	2	82	41%
(**8**)	25	5	na	49%
(**9**)	20	na	na	1.1 μΜ
**NDGA**	87	93	-	0.45 μM
**Trolox**	-	-	92	-

na: no activity was detected under the experimental conditions. * for compounds presenting promising anti-LOX activities, IC_50_ values are calculated additionally to the % inhibition.

**Table 3:** Binding affinity values (kcal/mol) for blind docking for all the synthesized compounds.

Compd.	Docking Scores (kcal/mol)
(**1**)	−5.2
(**2**)	−5.7
(**3**)	−7.2
(**4**)	−11.2
(**5**)	−8.7
(**6**)	−10.3
(**7**)	−8.4
(**8**)	−9.7
(**9**)	−10.6

## Data Availability

Data are contained within the article and Supplementary Materials.

## Supplementary Materials

The following supporting information can be downloaded at: https://www.mdpi.com/article/10.3390/molecules29061189/s1, ^1^H-NMR, ^13^C-NMR and Mass spectra of all the synthesized derivatives.
