# Supplementary material for: Synthesis and Antioxidant Activities of Novel Pyrimidine Acrylamides as Inhibitors of Lipoxygenase: Molecular Modeling and In Silico Physicochemical Studies
Source: Molecules. 2024 Mar 7;29(6):1189. doi: 10.3390/molecules29061189 (PMC10974930; doi:10.3390/molecules29061189)

Supporting information of the synthesized derivatives (**2**)-(**9**).

^{1}H NMR-**Compound 2**

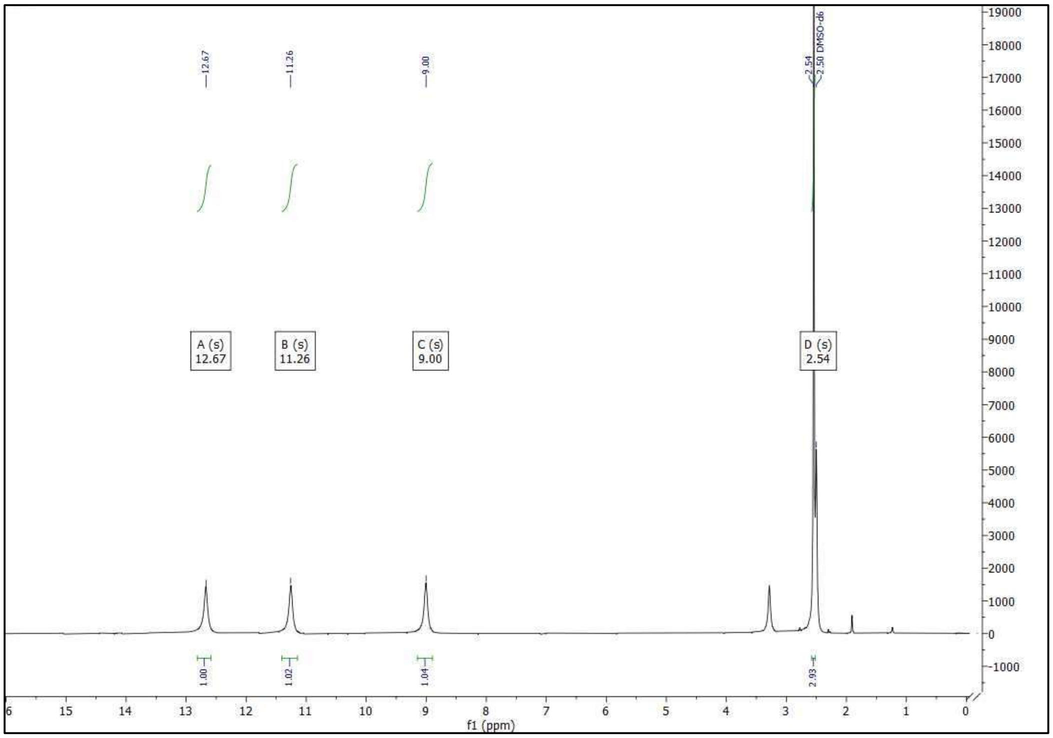

^{1}H NMR-**Compound 3**

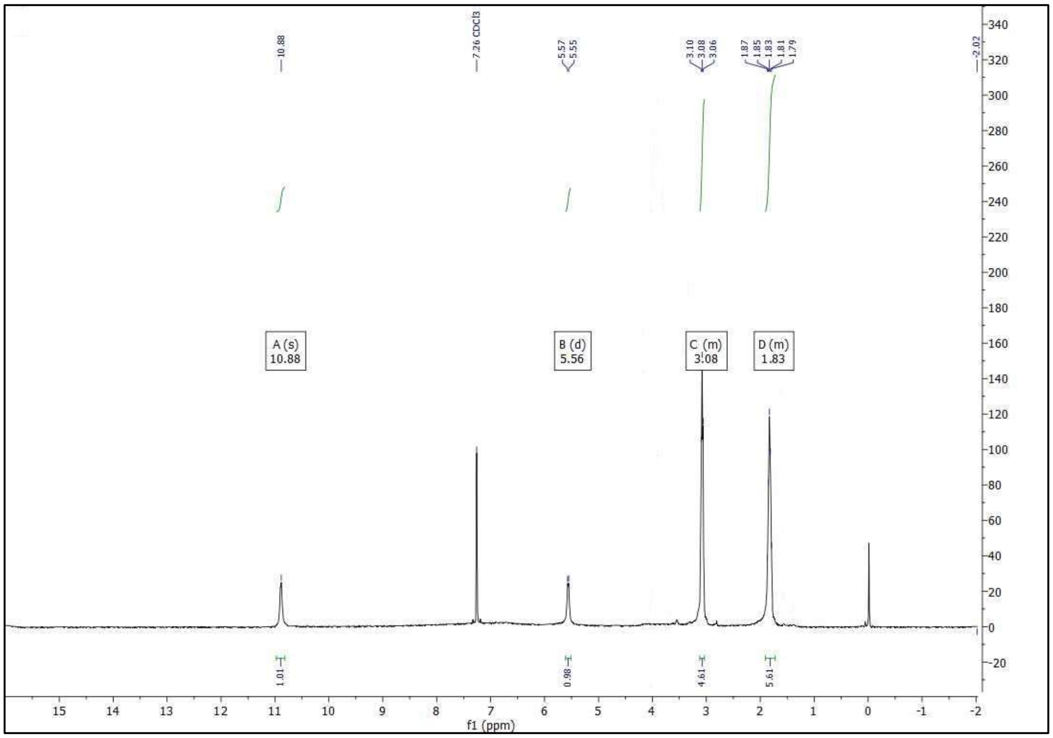

^{13}C NMR-**Compound 3**

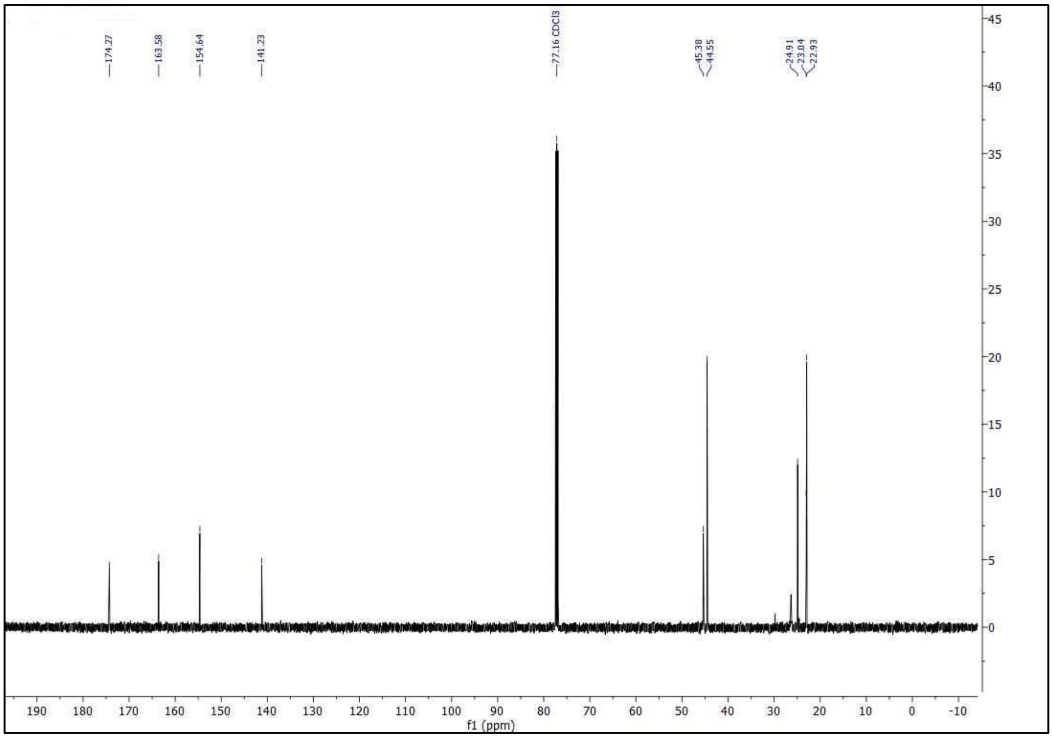

^{1}H NMR-**Compound 4**

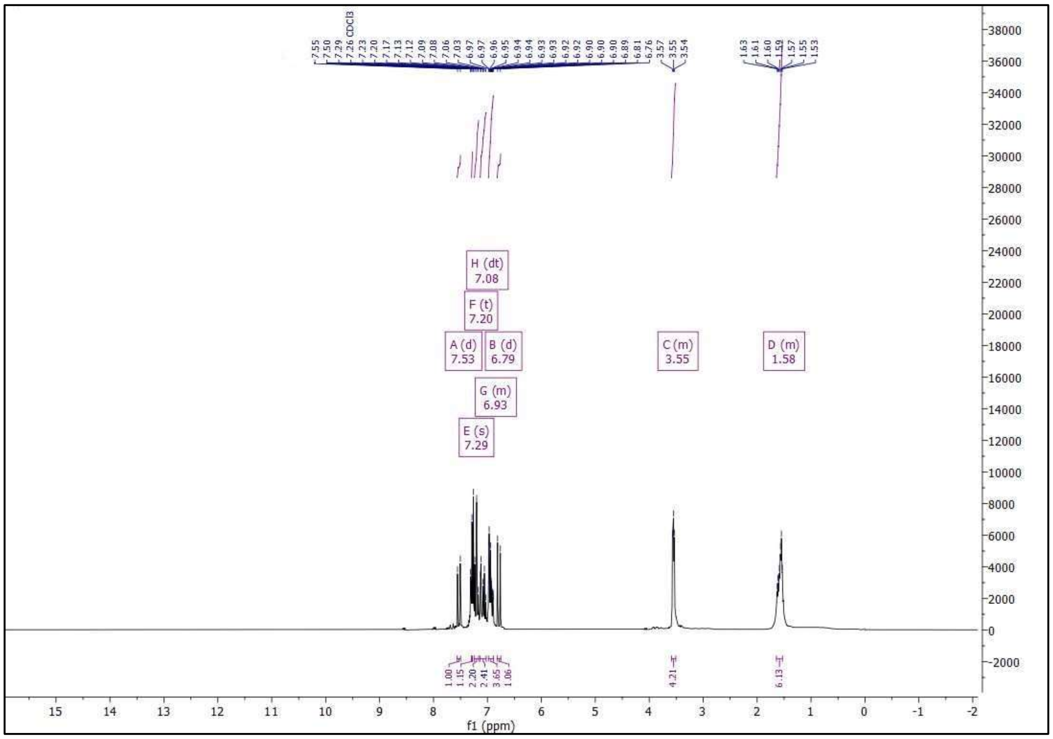

^{13}C NMR-**Compound 4**

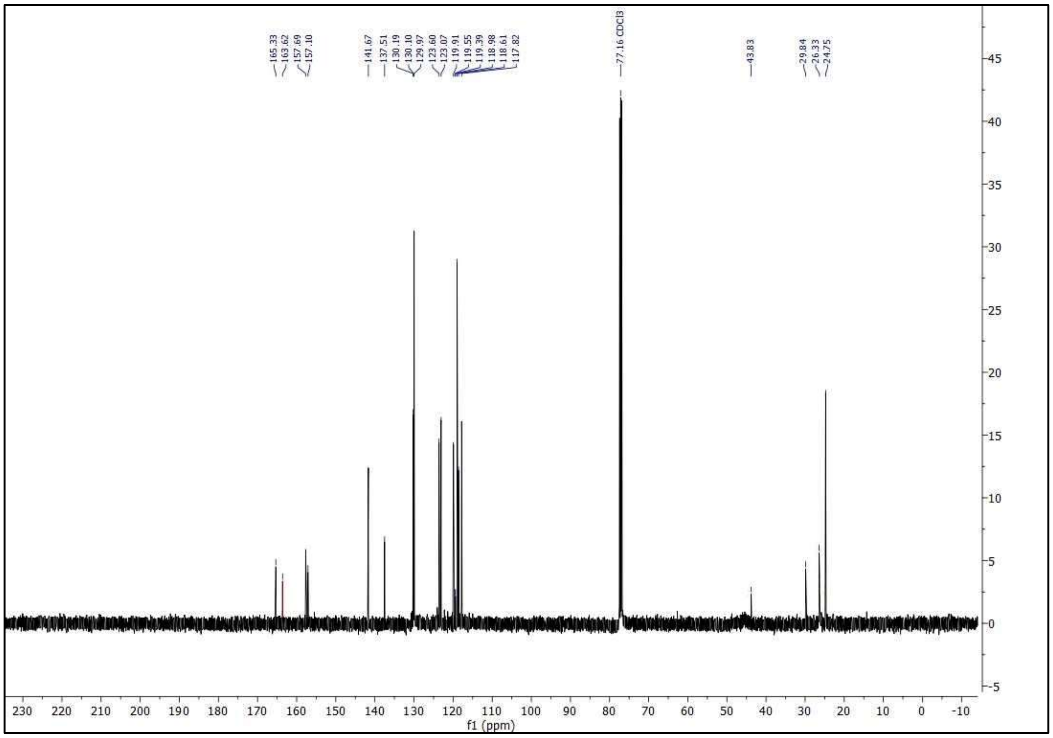

Mass Spectrum-**Compound 4 (**$C_{24}H_{23}N_5O_4$, MW: 445**)**

Positive

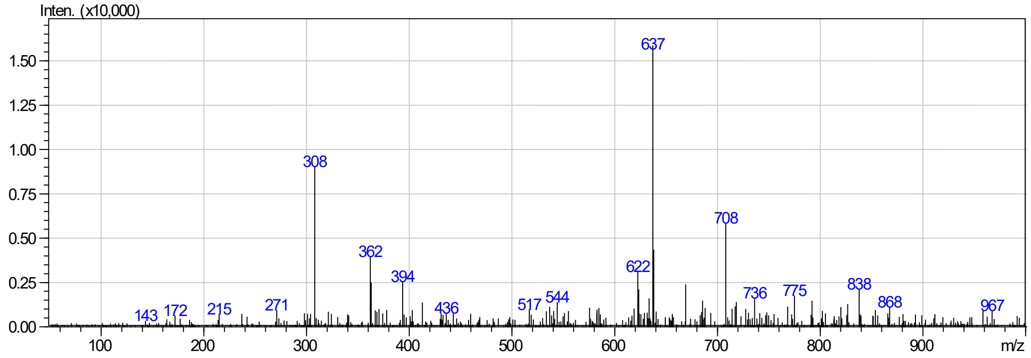

^{1}H NMR-**Compound 5**

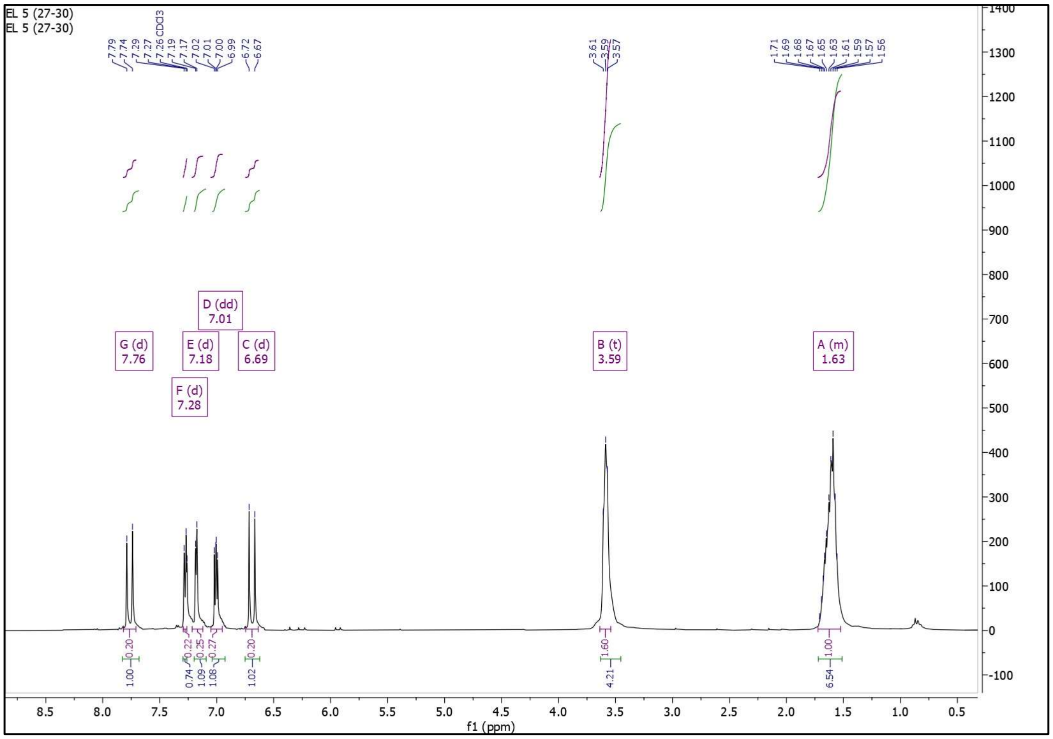

^{13}C NMR-**Compound 5**

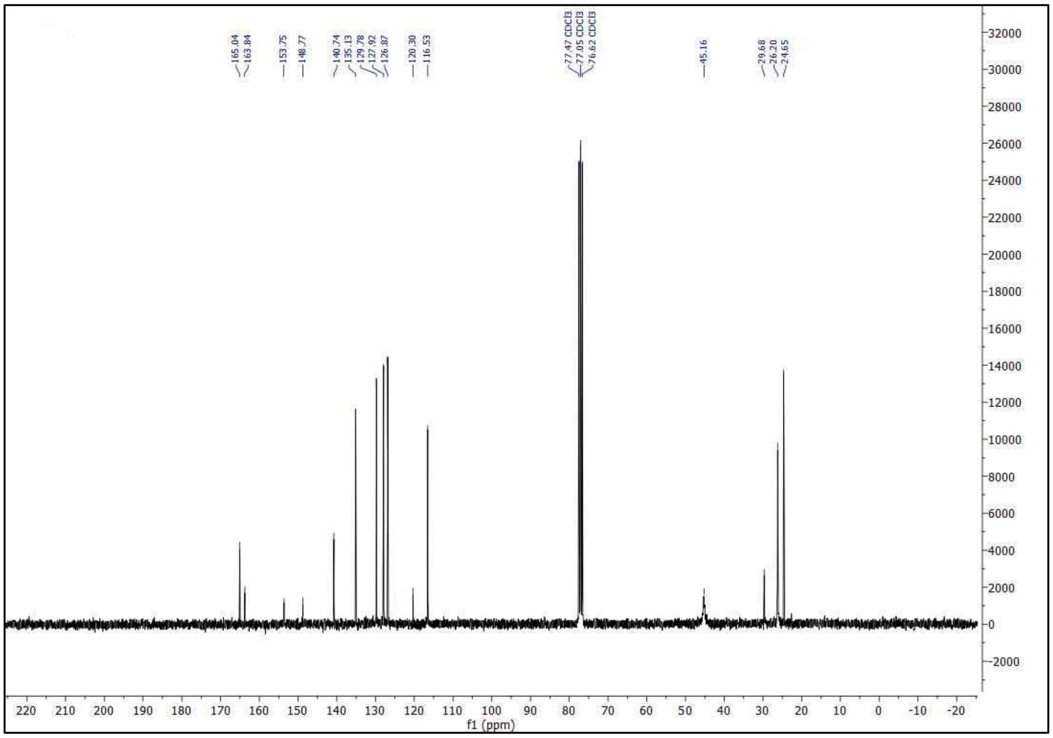

Mass Spectrum-**Compound 5** ($C_{16}H_{17}N_5O_3S$, MW: 359)

Positive

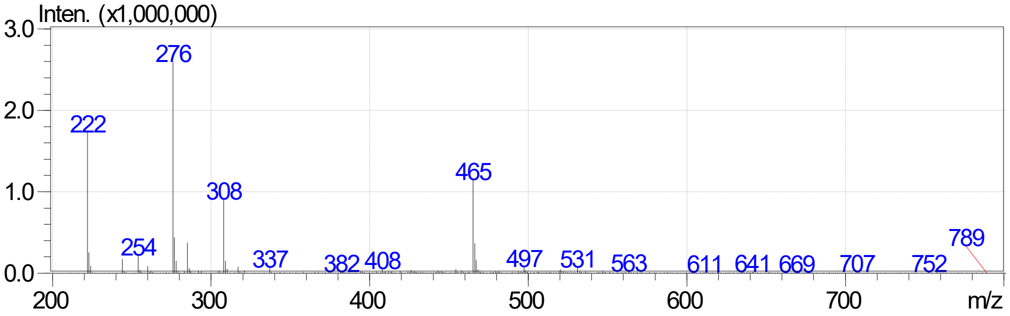

^{1}H NMR-**Compound 6**

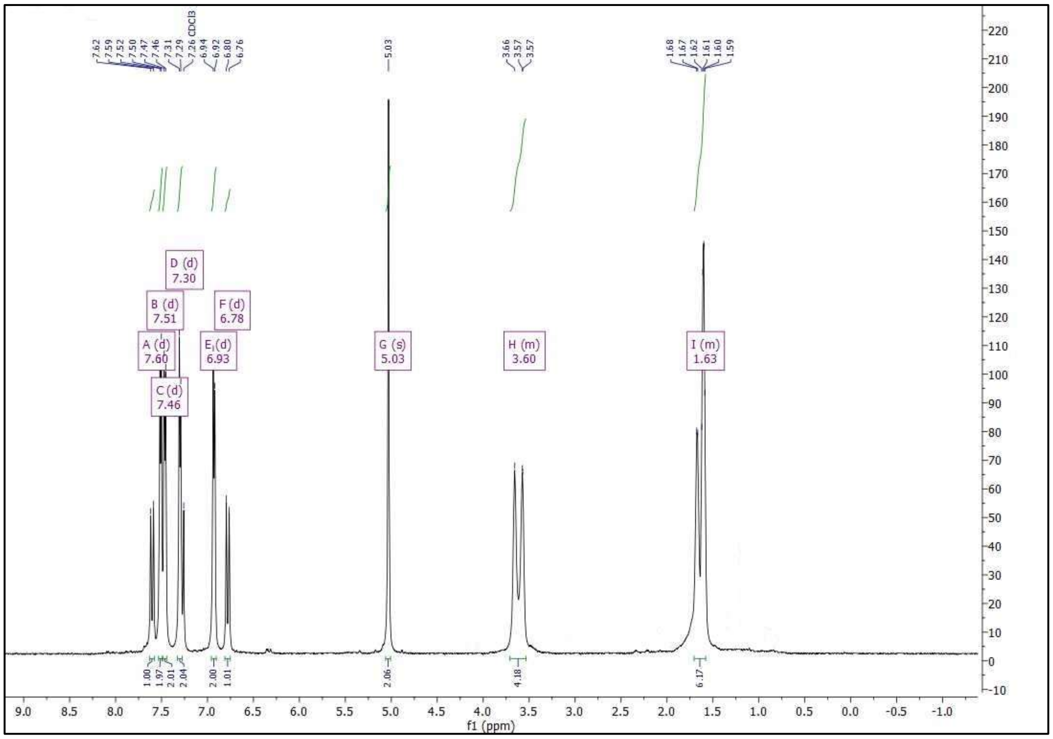

^{13}C NMR-**Compound 6**

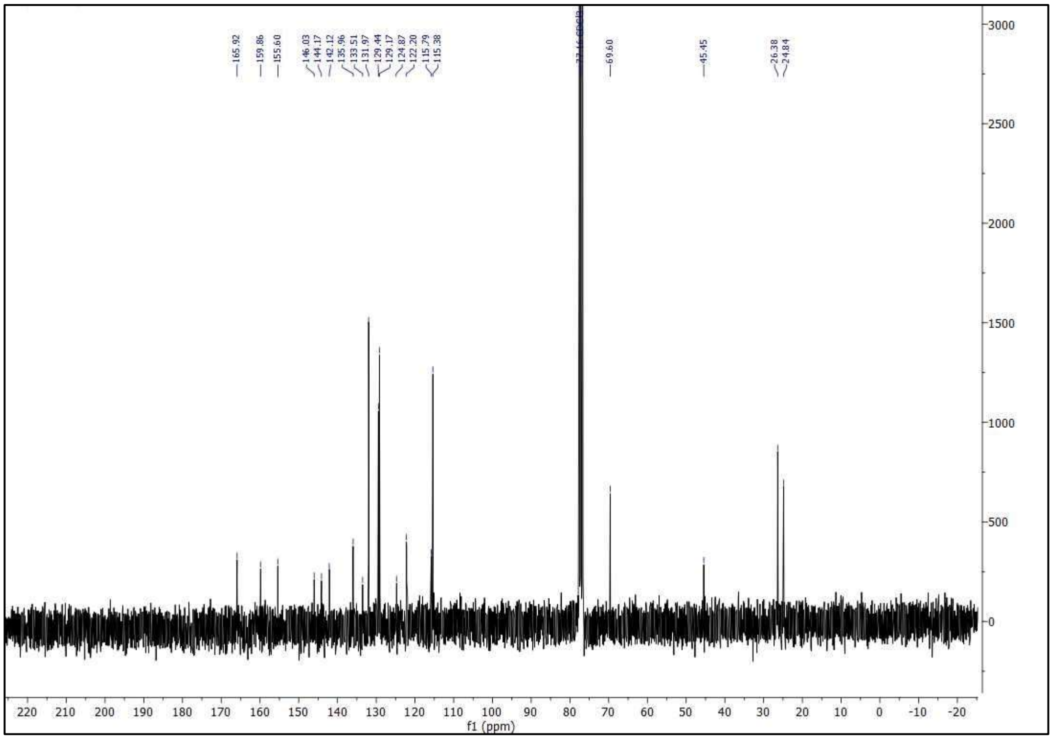

Mass Srectrum-**Compound 6 (**$C_{25}H_{24}BrN_5O_4$, MW: 538**)**

Positive

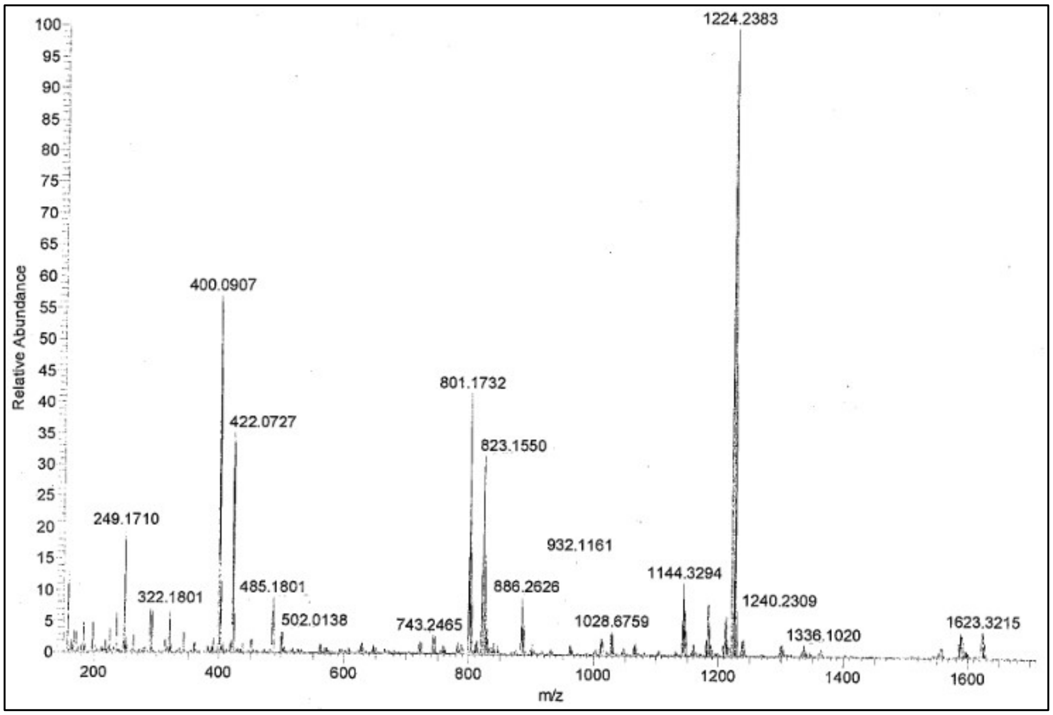

^{1}H NMR-**Compound 7**

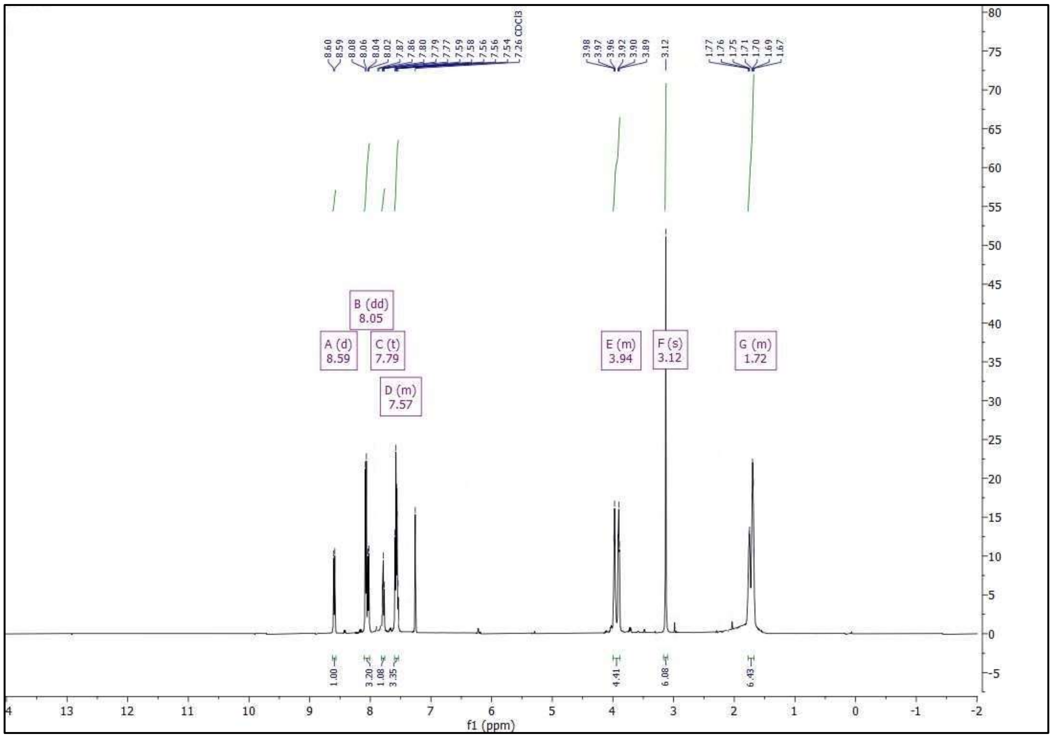

^{13}C NMR-**Compound 7**

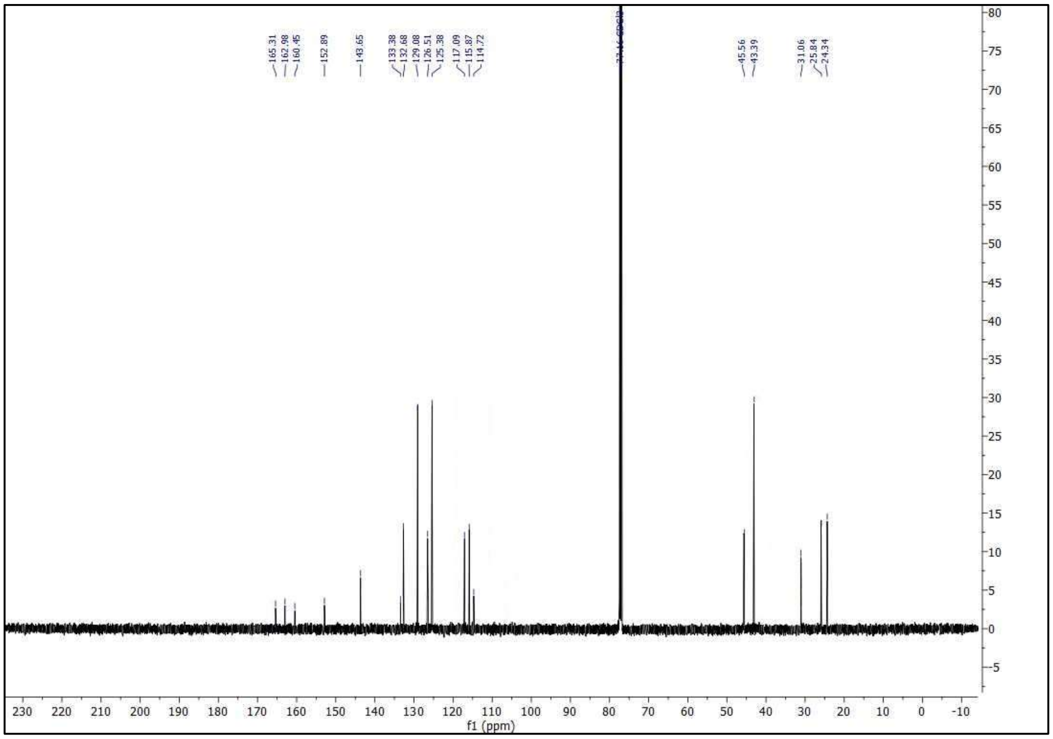

Mass Spectrum-**Compound 7 (**$C_{22}H_{26}N_6O_3$, MW: 422**)**
Positive

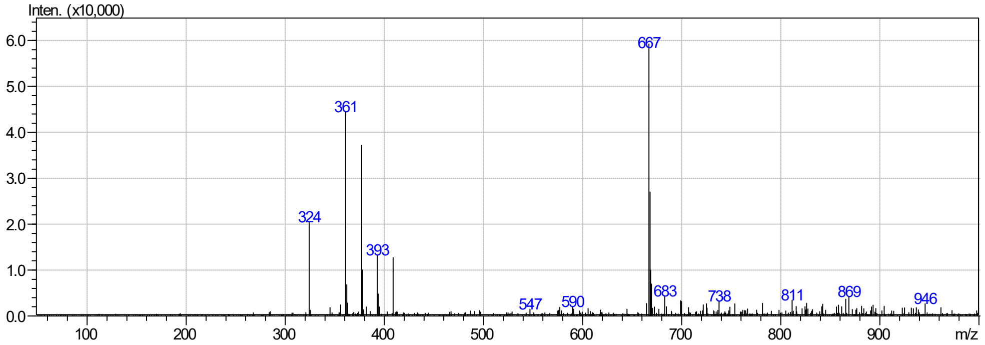

^{1}H NMR-**Compound 8**

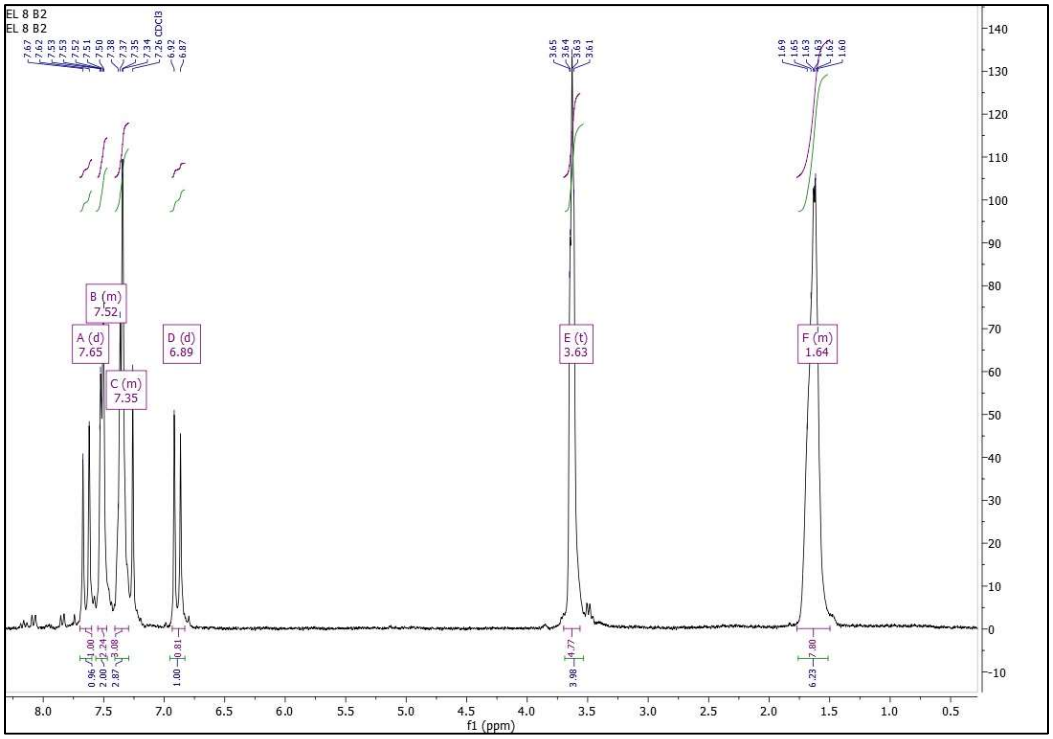

^{13}C NMR-**Compound 8**

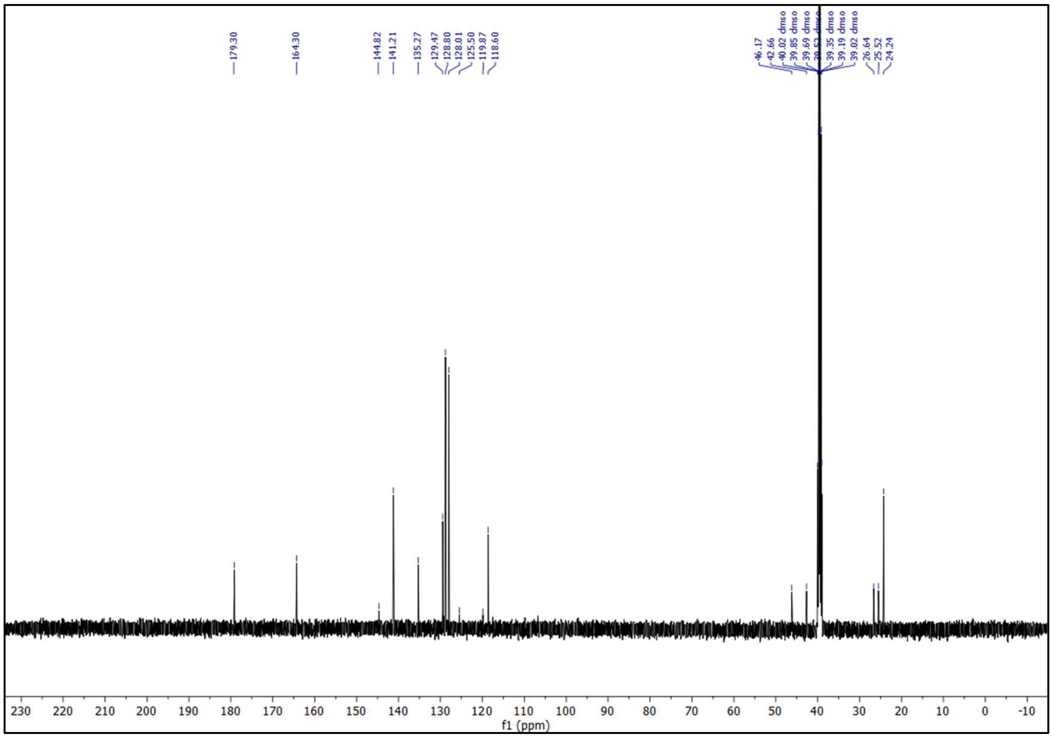

Mass Spectrum-**Compound 8** ($C_{18}H_{19}N_5O_3$, MW: 353)

Positive

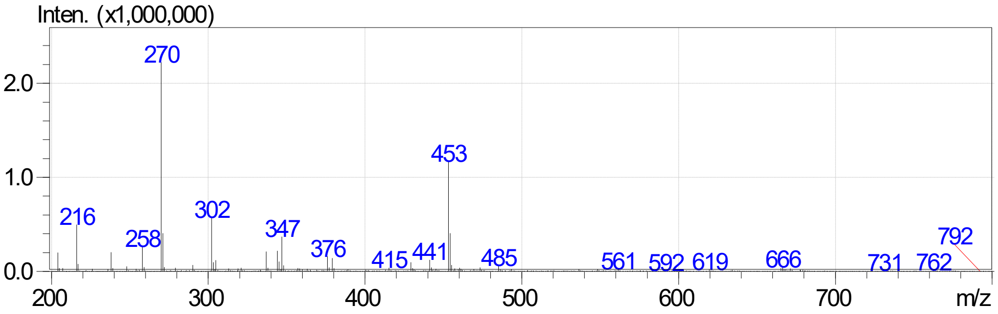

^{1}H NMR-**Compound 9**

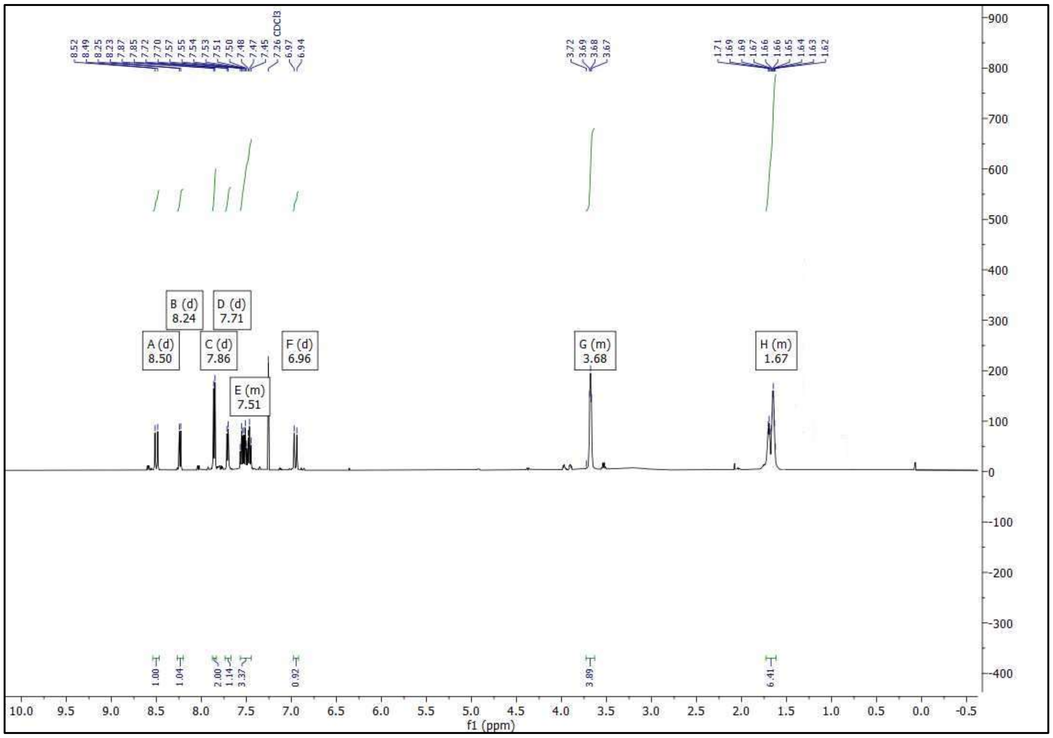

^{13}C NMR-**Compound 9**

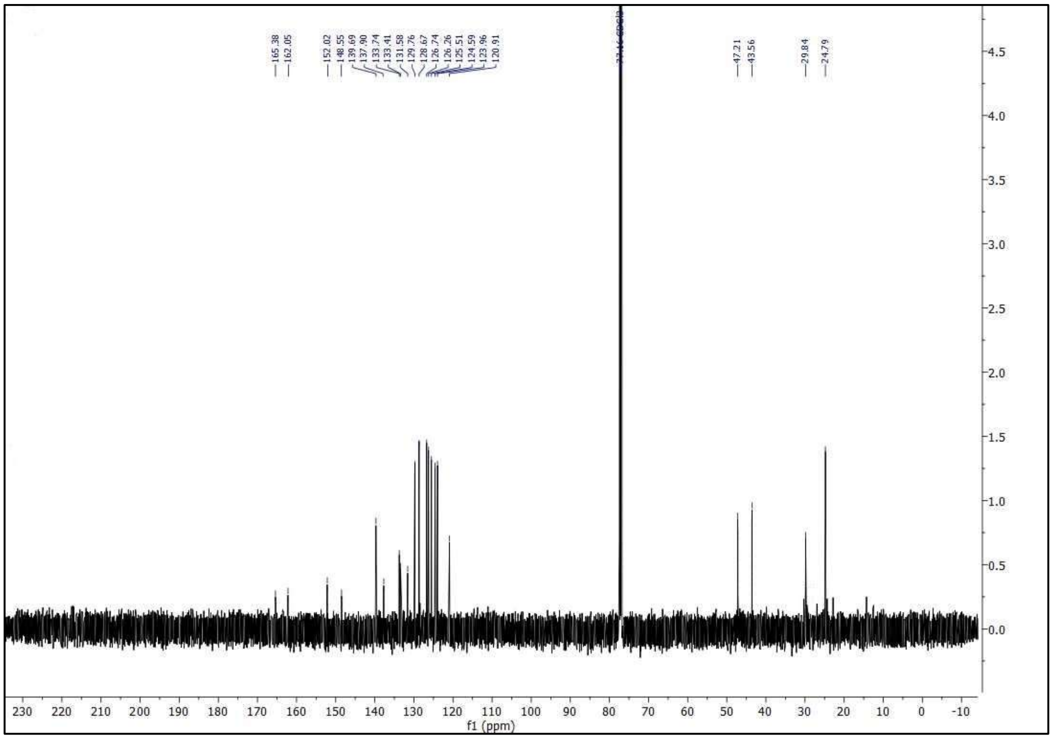

Mass Spectrum-**Compound 9** **(**$C_{22}H_{21}N_5O_3$, MW: 403**)**

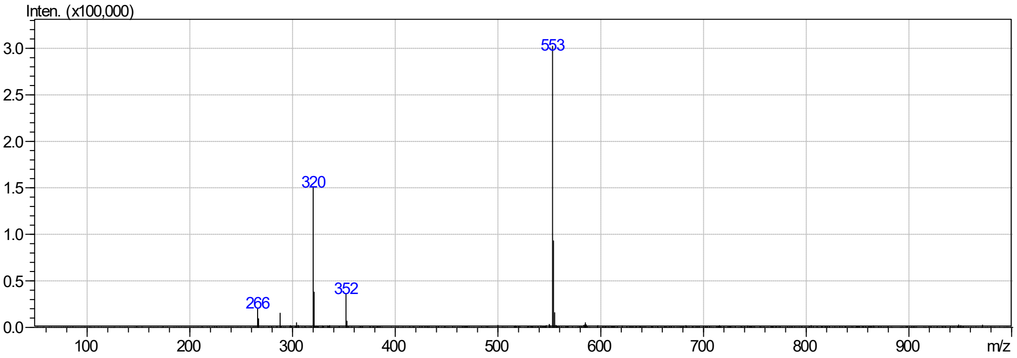

Supplement: Supplementary file 1 [file molecules-29-01189-s001.zip › molecules-2851751-supplementary.pdf]
